# Secondary metabolites isolated from *Fernandoa adenophylla* (Wall. ex G.Don) steenis as multitarget inhibitors of cholinesterases for the treatment of Alzheimer’s Disease, followed by molecular docking studies

**DOI:** 10.1371/journal.pone.0331119

**Published:** 2025-09-05

**Authors:** Maria, Asifullah Khan, Rahaf Ajaj, Abdur Rauf, Marryum, Zafar Ali Shah, Zubair Ahmad, Hassan A. Hemeg, Umer Rashid

**Affiliations:** 1 Department of Biochemistry, Abdul Wali Khan University, Mardan, Khyber Pakhtunkhwa, Pakistan; 2 Department of Environmental and Public Health, College of Health Sciences, Abu Dhabi University, Abu Dhabi, United Arab Emirates; 3 Department of Chemistry, University of Swabi, Swabi, Anbar, Khyber Pakhtunkhwa, Pakistan; 4 Department of Chemistry, COMSATS University Islamabad, Abbottabad Campus, Abbottabad, Pakistan; 5 Department of Agriculture Chemistry and Biochemistry, The University of Agriculture, Peshawar, Khyber Pakhtunkhwa, Pakistan; 6 Department of Medical Laboratory Technology, College of Applied Medical Sciences, Taibah University, Al-Medinah Al-Monawara, Madinah, Saudi Arabia; University of Rajshahi, BANGLADESH

## Abstract

Alzheimer’s disease (AD) is a neurodegenerative disorder categorized by the progressive loss of cognitive function, with acetylcholinesterase (AChE) and butyrylcholinesterase (BuChE) as key therapeutic targets. In this study, we report the isolation, characterization, and evaluation of the cholinesterase inhibitory potential of phytochemicals from *Fernandoa adenophylla* (Wall. ex G. Don) Steenis, a plant known for its medicinal properties. Using *in-vitro* enzyme inhibition assays, we identified five bioactive compounds, including lapachol (1), α-lapachone (2), peshawaraquinone (3), dehydro-α-lapachone (4), and an indanone derivative (5), which demonstrated significant inhibition of AChE and BuChE. The compounds exhibited varied inhibitory potency, with peshawaraquinone (3) showing the most promising AChE (IC_50_ = 0.90 ± 0.04 µM) and BuChE (IC_50_ = 8.39 ± 0.14 µM) inhibition, followed by dehydro-α-lapachone (4), which exhibited an AChE IC_50_ value of 2.64 ± 0.08 µM. Further, the selectivity index (SI) for AChE over BuChE was highest for dehydro-α-lapachone (SI = 21.1), suggesting its potential as a selective inhibitor. Molecular docking studies provided insights into the binding interactions between these compounds and the enzyme active sites, highlighting key interactions that may contribute to their inhibitory activity. These findings suggest that phytochemicals from *F. adenophylla* possess significant cholinesterase inhibition potential and may serve as leads for the development of novel therapeutic agents for Alzheimer’s disease.

## 1.0 Introduction

Medicinal plants are a rich source of bioactive secondary metabolites, having potential in healthcare, and have recently been regarded as novel pharmacophores in the production of modern medicines [1,2]. Plant-based remedies are used due to their low side effects, availability, and cost-effectiveness [3,4]. Compared to herbal products, synthetic drugs face bacterial resistance, which leads to ineffectiveness [5]. As a result, the application of phytochemical-based remedies is becoming more and more popular [6]. The development of robust ethnographic research is necessary for the identification and confirmation of therapeutic medicines derived from plants [7]. Current scientific research has focused on *Fernandoa adenophylla* (Wall. ex G.Don) Steenis, also known as *Heterophragma adenophyllum (*HA*)*, a member of the Bignoniaceae family native to both Southeast Asia and Africa, mainly in climatic conditions that have high temperatures. It is also known as Jiron, Dhopa-phali, and Karen wood. In traditional medicine, steenis is used to treat a variety of illnesses, including snake bites, skin diseases, seizures, and abscesses. [8,9]. It also relieves tense muscles by acting as a massage oil. *F. adenophylla* (Wall. ex G.Don) Steenis, is also used in traditional medicine, with its roots used to treat ulcers and eczema [10–12].

Phytochemical studies of *Fernandoa adenophylla* (Wall. ex G.Don) Steenis, have revealed the presence of various bioactive compounds [13]. Various phytochemicals have been isolated from *Fernandoa adenophylla* (Wall. ex G.Don) Steenis, and screened in vitro and in silico against various enzymes, targeting a wide range of proteins and pathways playing a wide role in the prevention of severe diseases [11,14].

Medicinal herbs have been the pillar of traditional healthcare systems for thousands of years and continue to be a significant source of novel therapeutic compounds. Ethnobotanical work has become more focused on documenting the medicinal and edible utilization of wild plant species. For instance, 92 wild plant species used as traditional food sources by locals in Bingöl, Turkey, that also possess reported medicinal applications were enumerated by Polat et al. (2017) [15]. Similarly, Babacan et al. (2022) conducted an ethno-veterinary survey in the same region and documented 46 plant species traditionally employed for the treatment of animal diseases, signifying the abundance of indigenous knowledge on plant therapeutics [16]. These findings underscore the global importance of wild medicinal plants and necessitate intensive systematic scientific exploration of their bioactive compounds. Accordingly, the current study investigates the cholinesterase inhibitory activity of phytochemicals isolated from Fernandoa adenophylla (Wall. ex G. Don) Steenis, a little-known medicinal plant, to contribute towards the discovery of potential candidates for the treatment of neurodegenerative disorders.

The main approach in this study is to treat Alzheimer’s disease (AD) by restoring the level of Ach and BuCh through the inhibition of cholinesterase [17]. Acetylcholinesterase (AChE, EC 3.1.1.7) and butyrylcholinesterase (BuChE, EC 3.1.1.8) are the two forms of cholinesterase enzymes that catalyze the hydrolysis of Ach and BuCh in choline and acetic acid [18]. AD is a neurological disease that primarily affects a patient’s social life due to a steady decline in memory and other cognitive abilities [19,20]. The illnesses also include the rupture of cholinergic pathways in the cerebral cortex and forebrain, associated with reduced levels of the neurotransmitters acetylcholine and butyrylcholine [21]. To treat the disease is to restore the level of Ach and BuCh through the inhibition of cholinesterase [22].

While inhibitors of acetylcholinesterase (AChE) and butyrylcholinesterase (BuChE) were among the first treatments approved for Alzheimer’s disease (AD), with clinical trials consistently documenting modest but real cognitive and functional improvements, the focus turned to Aβ- and tau-directed treatment strategies. However, continued failure of Aβ-directed clinical trials has cast considerable skepticism on this disease-modifying strategy [23]. In contrast, cholinesterase inhibitors are the only agent class to have had recurrent symptomatic efficacy in some well-controlled trials and have remained approved by international regulatory authorities [24]. Growing insight into their long-term cognitive benefits has renewed enthusiasm for examining other cholinesterase-active substances, most significantly natural phytochemicals, as presumably safer and equally effective symptomatic treatments. Natural products remain a bountiful source of structurally varied compounds for such therapeutic approaches. To this end, *Fernandoa adenophylla* (Wall. ex G.Don) Steenis, the non-famous plant of limited phytochemical and pharmacological research, possesses the potential to yield new novel scaffolds for potential cholinesterase inhibitors.

Thus, the aim of this study was to identify new potential molecules against acetylcholinesterase and butyrylcholinesterase using in silico tools and in vitro enzymatic inhibition. A combination of in silico and in vitro investigations is a viable technique for finding novel drug candidates targeting cholinesterases associated with Alzheimer’s. To the best of our knowledge, this article reports the cholinesterase inhibitory activity of secondary metabolites isolated from Fernandoa adenophylla (Wall. ex G.Don) Steenis. Although previous research has explored its antimicrobial and antioxidant activity, none of them has explored its area of application in the modulation of cholinergic enzymes related to neurodegenerative diseases, thereby reflecting the novelty of our work.

## 2.0 Materials and methods

### 2.1 Plant collection

The roots of *Fernandoa adenophylla* (Wall. ex G.Don) Steenis were collected from the Khyber Pakhtunkhwa region of Pakistan. The collection of this species for academic research is allowed under local regulations. The plant specimen was taxonomically identified by an expert from the Department of Botany, Abdul Wali Khan University Mardan (AWKUM).

### 2.2 Extraction and Isolation

The collected roots were shade-dried for 40–45 days, then ground into a fine powder. The powdered material was subjected to extraction with methanol using a Soxhlet apparatus for 6 hours. The resulting crude methanolic extract was fractionated based on polarity to facilitate the isolation of individual phytochemicals. Purification of the extract was carried out using column chromatography, which led to the isolation of five distinct compounds **(1–5)**. Column chromatography was performed using a silica gel column (Merck, Cat. No. 1.09385.1000, particle size 60–120 mesh). These compounds were further purified by sequential washing with both polar and non-polar solvents to ensure purity. The purified compounds were identified based on their chromatographic behavior and spectral data.

### 2.3 Characterizations of isolated compounds

Our research group previously elucidated the chemical structures of isolated compounds using advanced spectroscopic techniques such as H^1^-NMR, C^13^-NMR, 2D NMR, and mass spectrometry [25]. ^1^H and ^13^C NMR spectra were recorded on a Bruker Avance III HD 400 MHz spectrometer. The structural determination of all structures was carried out using ^1^H NMR and ^13^C NMR. The H^1^ NMR and C^13^ NMR spectra were obtained by a Varian Unity plus 300 apparatus using different solutions as internal references. The chemical shift values were measured in parts per million (ppm) about the coupling constants (J) in Hertz (Hz). High resolution mass spectra will be obtained from a Fourier transform ion cyclotron resonance (FT-ICR) mass spectrometer.

**Fig 1:**
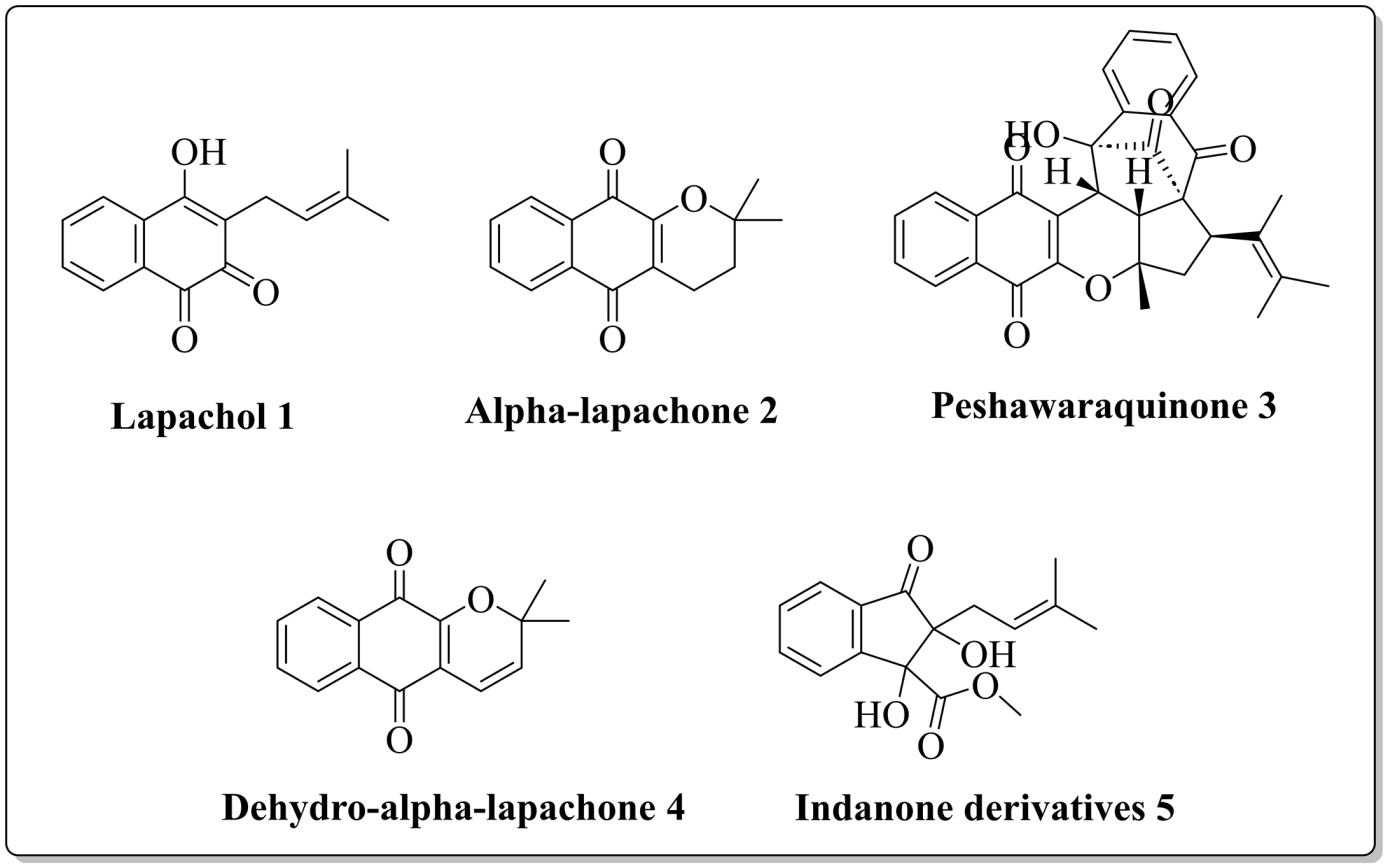
Structure of isolated compounds.

The structure of the isolated compounds (Lapachol 1, Alpha-lapachone 2, Peshawaraquinone 3, Dehydro-α-lapachone (4), Indanone derivatives 5) is provided in the inset of Fig 1.

### 2.4 *In-vitro* screening

The alcoholic extract (MeOH) and isolated compounds (**1–5**) from *F. adenophylla*, were assessed for *in-vitro* inhibitory activity against acetylcholinesterase (AChE) and butyrylcholinesterase (BuChE), following previously reported procedures [26,27].

#### 2.4.1 Cholinesterase inhibition assay using Ellman’s method.

The cholinesterase inhibitory activity of purified compound (**1–5**) were evaluated using Ellman’s colorimetric method. Enzymes from *Electrophorus electricus* (eeAChE) (Sigma-Aldrich, Cat. No. C3389) and equine serum (*Equus caballus*) BuChE (eqBuChE) (Sigma-Aldrich, Cat. No. C3389) were used [28]. Stock solutions of the test compounds were prepared in potassium phosphate buffer (0.1 M, pH 8.0). The reaction mixture consisted of Ellman’s reagent (5,5′-dithiobis (2-nitrobenzoic acid), DTNB), enzyme (0.03 U/mL of eeAChE or eqBuChE), and the test compound. Pre-incubation was performed at 30°C for 10 minutes, followed by the addition of 1 mM acetylthiocholine iodide (ATCI) or butyrylthiocholine iodide (BTCI) as substrates. The reaction mixture was further incubated for 15 minutes [29]. Enzyme activity was determined by measuring absorbance at 412 nm using a microplate spectrophotometer. All assays were conducted in triplicate. IC_50_ values were calculated by nonlinear regression of compound concentration versus percentage inhibition. The methodology ensured high reproducibility and accuracy in evaluating cholinesterase inhibition.

### 2.5 Molecular docking

Molecular docking was conducted to gain insights into the binding interactions and inhibitory mechanisms of the isolated compounds against AChE and BuChE. The docking studies were carried out using the MOE (Molecular Operating Environment, version 2019.01; Chemical Computing Group, Montreal, Canada) and Discovery Studio Visualizer (version 2020; BIOVIA, Dassault Systèmes, San Diego, USA) software suite. The crystallographic structure of targets was retrieved from the Protein Data Bank (https://www.rcsb.org) base in the PDB format with PDB accession codes 2CKM for AChE and 4BDS for BuChE. Protein structures were prepared using MOE’s structure preparation module, which involved 3D protonation and energy minimization to achieve a stable conformation. Ligand structures were also energy-minimized and protonated using default MOE settings. A maximum of 10 conformations were generated for each ligand. Based on docking score, the top-ranked conformations were selected for further analysis. Protein–ligand interactions were visualized using Discovery Studio, allowing interpretation of key binding residues and interaction types [30].

### 2.6 Statistical analysis

All experiments were performed in triplicate, and the results are expressed as SEM. IC_50_ values were determined using nonlinear regression analysis (GraphPad Prism). All statistical analyses were performed using GraphPad Prism version 9.5.1 (GraphPad Software, San Diego, CA, USA).

## 3.0 Results

### 3.1 *In-Vitro* cholinesterase inhibition assay of isolated compounds

The inhibitory activity of five isolated compounds was evaluated against two key cholinesterase enzymes: equine acetylcholinesterase (*ee*AChE) and equine butyrylcholinesterase (*eq*BuChE). The half-maximal inhibitory concentration (IC_50_) values were calculated and expressed as mean ± standard error of the mean (SEM). Additionally, the selectivity index (SI) was calculated as the ratio of IC_50_ values for eqBuChE to eeAChE, providing insight into the compounds’ preferential enzyme inhibition as shown in the inset of Table 1. Lapachol (1) exhibited strong inhibition against *ee*AChE (IC_50_ = 5.04 ± 0.24 µM) and weak inhibition against *eq*BuChE (IC_50_ = 68.34 ± 1.06 µM), resulting in a selectivity index (SI) of 13.6, suggesting a notable preference for AChE over BuChE. α-Lapachone (2) moderately inhibited both enzymes with IC_50_ values of 10.98 ± 0.97 µM (*ee*AChE) and 39.11 ± 0.97 µM (*eq*BuChE), yielding an SI of 3.6. Peshawaraquinone (3) was the most potent eeAChE inhibitor among all tested compounds, with an IC_50_ of 0.90 ± 0.04 µM. It also showed moderate inhibition of eqBuChE (IC_50_ = 8.39 ± 0.14 µM), resulting in a selectivity index of 9.3. Dehydro-α-lapachone (4) showed excellent inhibitory activity against *ee*AChE (IC_50_ = 2.64 ± 0.08 µM) and moderate inhibition of *eq*BuChE (IC_50_ = 55.58 ± 1.41 µM), yielding the highest selectivity index of 21.1, indicating exceptional preference towards AChE. Indanone derivative (5) displayed poor inhibition of *ee*AChE (IC_50_ = 53.21 ± 1.29 µM) but comparatively better activity against *eBuChE* (IC_50_ = 24.18 ± 1.04 µM), giving a low SI of 0.45, suggesting a preference for BuChE over AChE. These findings highlight the diverse inhibitory profiles and enzyme selectivity of the tested compounds. Notably, Dehydro-α-lapachone (4) exhibited the highest selectivity for AChE (SI = 21.1), followed by Lapachol (1) (SI = 13.6). In contrast, Indanone derivative (5) showed inverse selectivity, favoring BuChE. Despite not having the highest SI, Peshawaraquinone (3) was the most potent AChE inhibitor and stands out as a promising lead compound for further neuroprotective or anti-Alzheimer’s investigation.

**Table 1:** *In-vitro* cholinesterase inhibitory activity (IC_50_ ± SEM in µM) and selectivity index (SI) of isolated compounds.

		IC_50_ (μM) ± SEM^a^		
No.	Compound Name	(*ee*AChE)	*eq*BuChE	Selectivity Index (SI)
1	**Lapachol (1)**	5.04 ± 0.24	68.34 ± 1.06	13.6
2	**α-Lapachone (2)**	10.98 ± 0.97	39.11 ± 0.97	3.6
3	**Peshawaraquinone (3)**	0.90 ± 0.04	8.39 ± 0.14	9.3
4	**Dehydro-α-lapachone (4)**	2.64 ± 0.08	55.58 ± 1.41	21.1
5	**Indanone derivative (5)**	53.21 ± 1.29	24.18 ± 1.04	0.45
6	**Donepezil**	0.052 ± 0.002	5.41 ± 0.09	104

^***a***^ ± SEM represents mean values; **n* *= 3. ^**b**^SI = IC_50_ of BChE/IC_50_ of AChE.

**Fig 2:**
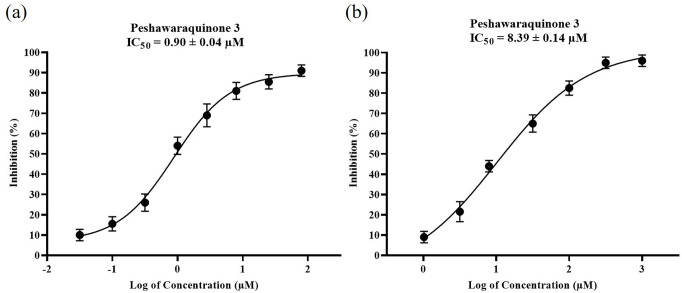
Dose-response curves of Peshawaraquinone-3 against (a) *Electrophorus electricus* acetylcholinesterase (eeAChE) and (b) equine serum butyrylcholinesterase (eqBuChE). The compound showed concentration-dependent inhibition of both enzymes. Values are expressed as mean ± SEM of three independent determinations. IC_50_ values were calculated using non-linear regression analysis (GraphPad Prism v9.5.1).

To further characterize the inhibitory profile of Peshawaraquinone (3) identified as the most potent AChE inhibitordose-response curves were generated for two enzymes. The results are graphically represented in Fig 2. Fig 2a, Peshawaraquinone-3 exhibited potent, concentration-dependent inhibition of equine acetylcholinesterase (*ee*AChE), with an IC_50_ value of 0.90 ± 0.04 µM, highlighting its strong binding affinity and high efficacy in blocking AChE activity.

In contrast, Fig 2b displays a moderate inhibitory effect on equine butyrylcholinesterase (*eq*BuChE), where the compound demonstrated an IC_50_ of 8.39 ± 0.14 µM. This reflects a clear preference for AChE over BuChE, further supported by its selectivity index (SI) of 9.3.

### 3.2 Molecular docking

#### 3.2.1 Docking analysis of AChE.

**Fig 3:**
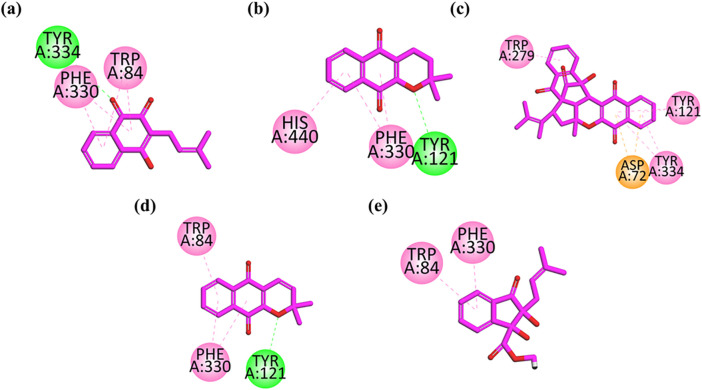
2D interaction plots of (a) Lapachol-1, (b) alpha lapachone-2, (c) Peshawaraquinone-3, (d) Dehydro-alpha-lapachone-4, (e) Indanone derivatives-5 in AChE. **Green lines** represent conventional hydrogen bonds, **pink lines** indicate π–π stacking interactions, and **yellow lines** denote π–sulfur interactions.The docking was performed using MOE 2019.01 and visualized with Discovery Studio Visualizer 2020.

To understand the binding interactions and affinities of the isolated compounds with acetylcholinesterase (AChE), molecular docking studies were conducted. The AChE active site comprises critical regions: the catalytic anionic site (CAS), including Trp84, Glu199, and Phe330, and the peripheral anionic site (PAS) involving Trp279, Tyr70, and Asp72. Additional residues such as Gly118, Gly119, Ala201, Trp233, Phe290, and Phe331 occupy the mid-gorge and contribute to ligand recognition and stabilization [31]. Fig 3 shows 2D interaction plots for all five compounds docked into AChE, revealing how each molecule interacts with the enzyme’s active residues. As shown in Fig 3a, Lapachol-1 exhibits strong π–π stacking interactions with Trp84 and Phe330, both located in the CAS. A stabilizing hydrogen bond with Tyr334 is also observed. These interactions suggest a high binding affinity, correlating with the compound’s strong inhibitory effect on AChE. Similarly, Fig 3b shows that Alpha-lapachone-2 also binds within the CAS, forming π–π stacking interactions with Phe330 and His440, alongside a polar interaction with Tyr121, which helps to position the ligand stably within the pocket. These contacts explain its moderate activity observed in vitro. Peshawaraquinone-3 forms key π–π interactions with Trp279, Tyr121, and Tyr334, as well as a π–sulfur interaction with Asp72 in the PAS as illustrated in Fig 3c. These multiple contacts anchor the ligand effectively across the gorge, supporting its superior inhibitory activity and selectivity towards AChE. Fig 3d shows that Dehydro-alpha-lapachone-4 is stabilized through π–π stacking interactions with Trp84 and Phe330, along with a polar interaction with Tyr121. These interactions contribute to its high selectivity and binding strength within the AChE binding pocket. Indanone derivative-5 engages in π–π stacking with Trp84 and Phe330, the key CAS residues, as demonstrated in the inset of Fig 3e.

So, these docking studies revealed that π–π stacking and hydrogen bonding are the primary stabilizing forces guiding these ligands into the AChE active site. Among all, Peshawaraquinone-3 displayed the most extensive interaction network, aligning with its strong in vitro inhibitory profile and highlighting its potential as a promising AChE inhibitor.

#### 3.2.2 Docking studies of BuChE.

To understand the molecular basis of ligand interactions with BuChE, docking studies were conducted using the isolated compounds. The active site of BuChE is formed by critical residues His438, Ser198, and Glu325, which make up the catalytic triad, and Tyr332 and Trp82, which contribute to ligand stabilization through hydrophobic and π-interactions.

**Fig 4:**
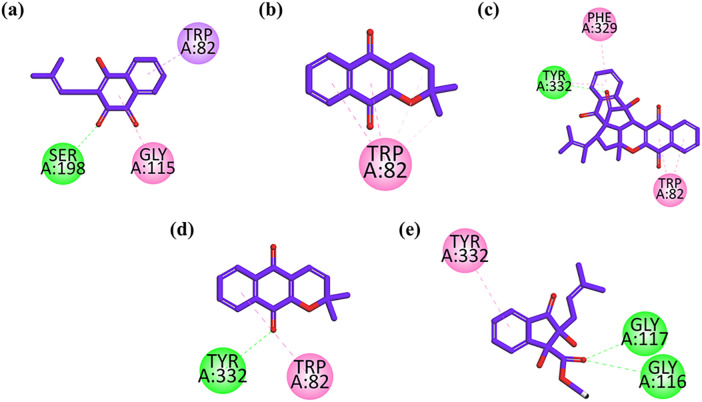
2D interaction plots of (a) Lapachol-1 (b) alpha lapachone-2 (c) Peshawaraquinone-3 (d) Dehydro-alpha-lapachone-4 (e) Indanone derivatives-5 in BuChE. The interaction map displays key binding features: **green lines** indicate conventional hydrogen bonding, **pink lines** represent π–π interactions, and **purple lines** correspond to π–σ interactions between the ligand and active site residues. The docking was performed using MOE 2019.01 and visualized with Discovery Studio Visualizer 2020.

Fig 4 illustrates 2D interaction plots showing the molecular interactions between each compound and the BuChE binding site. As shown in Fig 4a, Lapachol-1 binds to the enzyme via a π–σ interaction with Trp82, a key residue in the active pocket. Additionally, a π–π stacking interaction with Gly115 and a hydrogen bond with Ser198 contribute to its binding affinity. These contacts stabilize the ligand and explain its measurable inhibitory effect against BuChE. Similarly, Fig 4b shows the binding of Alpha-lapachone-2, which establishes strong π–π stacking interactions with Trp82. This interaction plays a key role in anchoring the compound in the enzyme’s pocket, suggesting it has good binding affinity despite lower selectivity compared to AChE. Peshawaraquinone-3 interacts with Trp82 and Phe329 through π–π stacking, and also forms a hydrogen bond with Tyr332, increasing its stability in the active site as depicted in Fig 4c. These multi-point interactions suggest that the compound is well accommodated in the BuChE pocket, supporting its moderate inhibitory potency observed experimentally. Dehydro-alpha-lapachone-4 binds BuChE through π–π stacking with Trp82 and forms a hydrogen bond with Tyr332, reinforcing its position in the catalytic site as shown in Fig 4d. These interactions help explain the compound’s moderate inhibition activity and indicate potential for future optimization. While in Fig 4e, Indanone derivative-5 shows strong π–π stacking with Tyr332 and forms hydrogen bonds with Gly116 and Gly117, contributing significantly to the ligand’s stabilization within the active site. Despite weaker AChE inhibition, these interactions suggest a preferential binding to BuChE.

The docking outcomes show that the isolated compounds form important contacts with essential residues in the active site of BuChE by mainly π–π stacking, and hydrogen bonding. The binding affinities deduced from the observed binding orientations indicate that the compounds are promising candidates for future study as inhibitors of BuChE.

### 3.3 Lipinski’s rule of five analysis

All five isolated compounds were evaluated for oral drug-likeness using Lipinski’s Rule of Five, which provides a predictive framework for assessing bioavailability based on key physicochemical properties (Table 2). Lipinski’s Rule of Five is used to assess the oral drug-likeness of a compound. It predicts whether a molecule is likely to be absorbed well in the human body based on:

**Table 2:** Lipinski’s Rule Parameters.

Parameter	Lapachol (1)	α-Lapachone (2)	Peshawaraquinone (3)	Dehydro-α-lapachone (4)	Indanone Derivative (5)
**Molecular Weight (Da)**	242.27	242.27	494.54	240.26	290.32
**LogP**	2.54	2.56	3.86	2.41	1.78
**H-bond Donors**	1	0	1	0	2
**H-bond Acceptors**	3	3	6	3	5
**Lipinski Rule Violations**	0	0	0	0	0

- Molecular weight < 500 Da
- LogP < 5
-  ≤ 5 hydrogen bond donors
-  ≤ 10 hydrogen bond acceptors
-  ≤ 1 violation of these rules

Compounds that comply with Lipinski’s rule are more likely to be bioavailable and orally active.

### 3.4 ADMET profiling

For the evaluation of the pharmacokinetic properties and safety profile of isolated compounds, ADMET parameters were in silico predicted using the admetSAR (classic) platform with emphasis on main absorption, distribution, metabolism, and toxicity markers important for CNS-active drug candidates.

As presented in the Table 3, the intestinal absorption was high for all compounds, and all were permeable through the blood–brain barrier (other than the indanone derivative), non-mutagenic, non-carcinogenic, and did not inhibit key CYP450 enzymes or P-glycoprotein, validating their promising pharmacokinetic and safety profiles.

**Table 3:** Key ADMET Parameters from admetSAR.

Parameter	Lapachol (1)	α-Lapachone (2)	Peshawaraquinone (3)	Dehydro-α-lapachone (4)	Indanone Derivative (5)
**Human Intestinal Absorption (HIA)**	Positive (1.0000)	Positive (1.0000)	Positive (0.9916)	Positive (1.0000)	Positive (0.9310)
**Blood–Brain Barrier (BBB) Penetration**	Positive (0.6401)	Positive (0.9312)	Positive (0.9035)	Positive (0.9007)	Negative (0.7548)
**AMES Toxicity**	Non-mutagenic (0.9133)	Non-mutagenic (0.7886)	Non-mutagenic (0.6924)	Non-mutagenic (0.7643)	Non-mutagenic (0.5000)
**Carcinogens**	Non-carcinogen (0.9150)	Non-carcinogen (0.9407)	Non-carcinogen (0.9348)	Non-carcinogen (0.9234)	Non-carcinogen (0.9446)
**CYP450 2D6 Inhibitor**	No (0.6167)	No (0.8056)	No (0.8027)	No (0.5000)	No (0.8579)
**CYP450 3A4 Inhibitor**	No (0.8820)	No (0.5174)	No (0.6756)	No (0.6466)	No (0.8729)
**P-glycoprotein Substrate**	No (0.6625)	No (0.7744)	No (0.7631)	No (0.6935)	No (0.8001)

## 4.0 Discussion

The aim of this study was to explore the potential of *Fernandoa adenophylla* (Wall. ex G.Don) Steenis, as a source of bioactive compounds for the inhibition of AChE and BuChE, two enzymes implicated in Alzheimer’s disease AD. The results of both in vitro enzyme inhibition assays and molecular docking studies have provided valuable insights into the inhibitory profiles of five isolated compounds from *Fernandoa adenophylla* (Wall. ex G.Don) Steenis, with a particular focus on their selective inhibition of AChE and BuChE. The integrity of the cholinesterase inhibition assay was confirmed by using Donepezil as a positive control. The IC_50_ values determined for Donepezil (AChE: 0.052 ± 0.002 µM, BuChE: 5.41 ± 0.09µM) agreed well with literature values, which ensured the precision and reliability of the assay conditions. The isolated compounds displayed varying degrees of inhibitory activity against both AChE and BuChE, with some showing remarkable selectivity towards AChE. Lapachol (1), for instance, exhibited a strong inhibitory effect against AChE (IC_50_ = 5.04 ± 0.24 µM) but only weak inhibition against BuChE (IC_50_ = 68.34 ± 1.06 µM), resulting in a selectivity index (SI) of 13.6. This high SI suggests that Lapachol preferentially targets AChE, making it a promising candidate for further investigation as an anti-Alzheimer’s agent. Similarly, Peshawaraquinone (3), the most potent AChE inhibitor (IC_50_ = 0.90 ± 0.04 µM), displayed a moderate inhibition against BuChE (IC_50_ = 8.39 ± 0.14 µM), with an SI of 9.3. This compound’s potent inhibition of AChE, coupled with its relatively lower activity against BuChE, further underscores its potential as a lead compound in the development of selective cholinesterase inhibitors for AD treatment. Dehydro-α-lapachone (4) showed the highest selectivity for AChE (SI = 21.1), demonstrating its exceptional preference for AChE inhibition. The compound’s IC_50_ value for AChE inhibition (2.64 ± 0.08 µM) and moderate activity against BuChE (IC_50_ = 55.58 ± 1.41 µM) suggest that it may be particularly effective in modulating cholinergic dysfunctions associated with AD. On the other hand, the Indanone derivative (5) showed a lower preference for AChE (IC_50_ = 53.21 ± 1.29 µM) and better inhibition of BuChE (IC_50_ = 24.18 ± 1.04 µM), with a SI of 0.45, indicating a potential preference for BuChE over AChE.

These findings are consistent with previous studies, even showing more potency than the previously published data in the inhibition of cholinesterases by naturally derived compounds, highlighting the importance of selectivity in cholinesterase-targeted therapies for AD [32]. The higher selectivity for AChE inhibition observed for some of the compounds is particularly promising, as AChE is a key target for the symptomatic treatment of AD. However, BuChE inhibition could also provide therapeutic benefits, especially considering its involvement in the progression of AD.

Molecular docking studies provided detailed insights into the interactions between the isolated compounds and the cholinesterase enzymes. The docking results revealed that π–π stacking interactions and hydrogen bonding play critical roles in stabilizing the compounds within the AChE and BuChE active sites. As a reference point for the molecular docking outcome, Donepezil was docked into the active sites of both acetylcholinesterase (AChE) and butyrylcholinesterase (BuChE) with the same docking protocol that was used with the test compounds. Donepezil in AChE engaged in interactions with essential catalytic residues like Ser203, His447, and Glu334, and also interacted with Trp86 and Phe338 within the anionic and peripheral anionic sites, as has been reported in previously published crystallographic data. Donepezil made hydrogen bonds and hydrophobic interactions with Ser198, His438, Glu325, Trp82, and Tyr332 in BuChE, which are essential for inhibiting the enzyme. Interestingly, a number of the isolated compounds had binding poses that were superimposed with the active or peripheral binding site of Donepezil, whereas others had different binding orientations, indicating the potential for good interactions and cholinesterase inhibition mechanisms.

Lapachol (1) formed π–π stacking interactions with Trp84 and Phe330, key residues in the AChE active site, and a stabilizing hydrogen bond with Tyr334, which correlates with its strong inhibitory effect on AChE. Similarly, Peshawaraquinone (3) exhibited extensive interactions with both the catalytic anionic site (CAS) and peripheral anionic site (PAS) of AChE, contributing to its superior binding affinity and potent inhibitory activity. The docking analysis of BuChE further revealed that these compounds also form important contacts with critical residues in the BuChE active site, such as Trp82 and Tyr332. Peshawaraquinone (3), for instance, interacted with Trp82 and Phe329 through π–π stacking and also formed a hydrogen bond with Tyr332, increasing its stability within the BuChE pocket.

This dual activity of Peshawaraquinone (3) in both AChE and BuChE inhibition highlights its potential as a dual-target agent, although its selectivity for AChE makes it a more promising candidate for AD therapy.

The docking results were in good agreement with the experimental in vitro data, where compounds such as Lapachol (1) and Dehydro-α-lapachone (4) exhibited high selectivity towards AChE. This further validates the use of molecular docking as a tool for predicting binding affinities and guiding the selection of compounds for further in vitro and in vivo testing.

The results of this study suggest that *Fernandoa adenophylla* (Wall. ex G.Don) Steenis, holds promise as a potential source of cholinesterase inhibitors, particularly for the treatment of Alzheimer’s disease. Compounds like Peshawaraquinone (3), Lapachol (1), and Dehydro-α-lapachone (4) exhibited strong and selective inhibition of AChE, which is a hallmark of many current AD therapies. Furthermore, the relatively low toxicity and the natural origin of these compounds make them attractive alternatives to synthetic drugs, which often suffer from undesirable side effects and resistance issues.

Although Peshawaraquinone (3) and Dehydro-α-lapachone (4) demonstrated promising results, further optimization of these compounds is required to enhance their bioavailability and therapeutic efficacy. Additionally, the selectivity of these compounds for AChE over BuChE is advantageous, as excessive BuChE inhibition could lead to unwanted side effects, such as the disruption of normal cholinergic function.

## 5.0 Conclusions

The study presents compelling evidence for the potential of *Fernandoa adenophylla* (Wall. ex G. Don) Steenis, as a source of novel cholinesterase inhibitors for Alzheimer’s disease. The isolated compounds, particularly Peshawaraquinone (3) and Dehydro-α-lapachone (4), demonstrated significant AChE inhibitory activity with good selectivity, making them promising candidates for future therapeutic development. Further in vivo studies and clinical trials are needed to validate their efficacy and safety profiles. The integration of *in-silico* and *in-vitro* approaches in this study offers a comprehensive strategy for identifying and optimizing bioactive compounds for neurodegenerative diseases, opening the door for the development of plant-based therapies for AD.
